# Characterization of the *WAK* Gene Family Reveals Genes for FHB Resistance in Bread Wheat (*Triticum aestivum* L.)

**DOI:** 10.3390/ijms23137157

**Published:** 2022-06-28

**Authors:** Xiaobo Xia, Xu Zhang, Yicong Zhang, Lirong Wang, Qi An, Qiang Tu, Lei Wu, Peng Jiang, Peng Zhang, Lixuan Yu, Gang Li, Yi He

**Affiliations:** 1 CIMMYT-JAAS Joint Center for Wheat Diseases, Key Laboratory of Germplasm Innovation in Downstream of Huaihe River (Nanjing), Ministry of Agriculture and Rural Affairs, Jiangsu Academy of Agricultural Sciences, Nanjing 210014, China; xiaxiaobo133@163.com (X.X.); xuzhang@jaas.ac.cn (X.Z.); zhangyicong0626@163.com (Y.Z.); lirong_wang0521@sina.com (L.W.); anqi9510@126.com (Q.A.); qiangtuswust@sina.com (Q.T.); wulei@jaas.ac.cn (L.W.); hmjp2005@163.com (P.J.); zhangpeng@jaas.ac.cn (P.Z.); lixuan_yu@sina.com (L.Y.); 2 College of Plant Protection, Nanjing Agricultural University, Nanjing 210095, China; 3 Wheat Research Institute, School of Life Sciences and Engineering, Southwest University of Science and Technology, Mianyang 621010, China

**Keywords:** wheat, wall-associated kinase, *TaWAK*, gene family, gene expression, Fusarium head blight, resistance

## Abstract

Wall-associated kinases (WAKs) are important receptor-like proteins that play major roles in plant defense against pathogens. Fusarium head blight (FHB), one of the most widespread and devastating crop diseases, reduces wheat yield and leads to quality deterioration. Although WAK gene families have been studied in many plants, systematic research on bread wheat (*Triticum aestivum*) and its role in FHB resistance, in particular, is lacking. In this study, we identified and characterized 320 genes of the TaWAK family in wheat distributed across all chromosomes except 4B and divided them into three phylogenetic groups. Duplication and synteny analyses provided valuable information on the evolutionary characteristics of the *TaWAK* genes. The gene expression pattern analysis suggested that *TaWAK* genes play diverse roles in plant biological processes and that at least 30 genes may be involved in the response to Fusarium infection in wheat spikes, with most of the genes contributing to pectin- and chitin-induced defense pathways. Furthermore, 45 *TaWAK* genes were identified within 17 hcmQTLs that are related to wheat FHB resistance. Our findings provide potential candidate genes for improving FHB resistance and insights into the future functional analysis of *TaWAK* genes in wheat.

## 1. Introduction

Plants have evolved to have two layers of innate immune systems to prevent or minimize the damage caused by pathogenic infection. This is also referred to as the “zig-zag” model, which includes pathogen-associated molecular pattern (PAMP)-triggered immunity (PTI), and effector-triggered immunity (ETI) [1]. PTI is moderated by numerous receptor-like kinases (RLKs) and receptor-like proteins that act as pattern recognition receptors and detect PAMPs as the first layer of inducible defense; this is also considered to confer broad-spectrum resistance to nonadapted potential pathogens [2]. Wall-associated kinases (WAKs) are a unique subfamily of RLKs that typically contain the extracellular WAK galacturonan-binding (GUB), extracellular epidermal growth factor (EGF), transmembrane (TM), and intracellular serine/threonine kinase domains [3]. The extracellular domain of WAKs is involved in cytoplasm–cell wall communication, whereas the intracellular kinase domain is concerned with the activation of cytoplasmic signaling cascades in plant defense reactions [4]. WAKs are the only type of receptors involved in cell-wall signaling in addition to their roles in the growth and development and in the tolerance to abiotic stresses. A growing number of studies have indicated that WAKs play key roles in plant defense against pathogens [5].

Data on plant WAKs and their roles in signal transduction and pathogen stress responses are derived mostly from studies on *Arabidopsis thaliana* [6]. *AtWAK1* was the first WAK-encoding gene identified in plants and was shown to bind with high affinity to pathogen- and damage-induced pectin fragments or oligogalacturonides, which have long been considered elicitors of plant defense [7,8]. Overall, 22 *WAK* genes have been detected in the Arabidopsis genome [3], and several studies have demonstrated that these are functional genes associated with pathogen defense. For example, *AtWAK1* confers enhanced resistance to the necrotrophic fungus *Botrytis cinerea* [9]; *AtWAKL22* plays a role in resistance to Fusarium wilt disease [10]; and *AtWAKL10* is responsible for resistance to *Pseudomonas syringae* [11]. Furthermore, WAKs play a critical role in pathogen responses in other plant species. In maize (*Zea mays*), *ZmWAK-RLK1* (encoded by *Htn1*) confers resistance to northern corn leaf blight and *ZmWAK qHSR1* enhances resistance to head smut [12,13]. In rice (*Oryza sativa*), *OsWAK1*, *OsWAK14*, *OsWAK91*, and *OsWAK92* positively regulate quantitative resistance to the blast fungus *Magnaporthe oryzae* [4,14]. Additionally, *Xa4* encodes a WAK that strengthens the cell wall and enhances the resistance to bacterial infection [15]. *SlWAK1* in tomatoes (*Lycopersicon esculentum*) promotes apoplastic immunity against the bacterial pathogen *Pseudomonas syringae* [16]. These findings assert that the *WAK* gene family is imperative for plant defense against pathogens.

Bread wheat (*Triticum aestivum*) is an important cultivated grain crop worldwide, and its products fulfill the food requirements of approximately 35% of the global population. Demands for crop production increase as the world’s population increases; however, pathogen-related diseases pose serious problems in agricultural production yield and quality [17]. Recently, several WAKs from wheat have been shown to be involved in defense against pathogens. The WAK-like protein Stb6 confers resistance to *Septoria tritici* blotch disease caused by *Zymoseptoria tritici* [18]. *TaWAK6* is involved in adult plant resistance to wheat leaf rust [19], and *TaWAK-6D* and *TaWAK7D* mediate resistance to *Fusarium pseudograminearum* and *Rhizoctonia cerealis* infections, respectively [20,21]. Among these crop pathogens, Fusarium head blight (FHB), mainly caused by *Fusarium graminearum (Fg)*, is a widespread and devastating crop disease. It affects small grain cereals and causes huge losses in grain yield and substantial deterioration in grain quality because of kernel contamination with harmful mycotoxins such as deoxynivalenol (DON). Although many quantitative trait loci (QTLs) associated with FHB resistance have been reported, only a few genes have been identified to confer FHB resistance [22]. For instance, *WAK2* isolated from the *QFhb.mgb-2A* region of durum wheat, and the *TaWAK2A-800* region of bread wheat is responsible for FHB resistance [23,24]. *WAKs* are now being recognized as playing important regulatory roles in plant immunity; therefore, the identification of new *WAK* genes that are critical for FHB responses facilitates the genetic manipulation of these candidates for improving FHB resistance in wheat breeding programs.

Genome sequences can provide valuable information for genome-based investigations. To date, the *WAK* gene family has been comprehensively identified in various plant species such as Arabidopsis [3], tomato [25], cotton [26], rice [27], barley [28], and walnuts [29]. However, systematic investigations of WAKs are yet to be performed in the wheat genome. The high-quality version of the hexaploid wheat genome assembled and annotated by the International Wheat Genome Sequencing Consortium allows the identification and characterization of WAK family members in wheat [30]. In this study, we identified and characterized the wheat *WAK* gene family comprising 320 genes in the bread wheat genome. A detailed characterization of gene structures, evolutionary relationships, expansion, expression levels, and subcellular localization of the wheat *WAK* genes is presented. Furthermore, online data were used to analyze the transcript accumulation of each family member in response to FHB, and the expression levels of 20 genes were evaluated after *Fg* infection and pectin inoculation via quantitative reverse transcription PCR (qRT-PCR). This study aimed to provide a global viewpoint for the molecular characterization of the *WAK* gene family in wheat and uncover new potential genes involved in FHB infection in crops so as to provide a basis for further functional studies and FHB-resistant wheat breeding.

## 2. Results

### 2.1. Genome-Wide Identification and Phylogenetic Analysis of the WAK Gene Family in Wheat

To characterize the putative WAKs in wheat, we performed a genome-wide analysis of the whole wheat genome with BLASTP [30] and identified the members of the WAK family using the AtWAK/AtWAKL proteins from Arabidopsis [31] as query sequences. All candidates filtered using the Pfam and SMART websites with three domains (WAK_GUB domain, TM domain, and protein kinase domain) were predicted to encode the WAK proteins. Overall, our analysis resulted in the identification of 320 *TaWAK* genes in wheat (Table S1). To analyze the gene characteristics, the encoded protein molecular weight (MW) and isoelectric points (pI) were predicted (Table S1). Among the 320 TaWAKs, TraesCS3D02G494200 had the highest MW of 119.2 kDa, whereas TraesCS5D02G374300 had the lowest MW of 65.4 kDa; the pI values ranged from 5.09 to 9.24.

**Figure 1:**
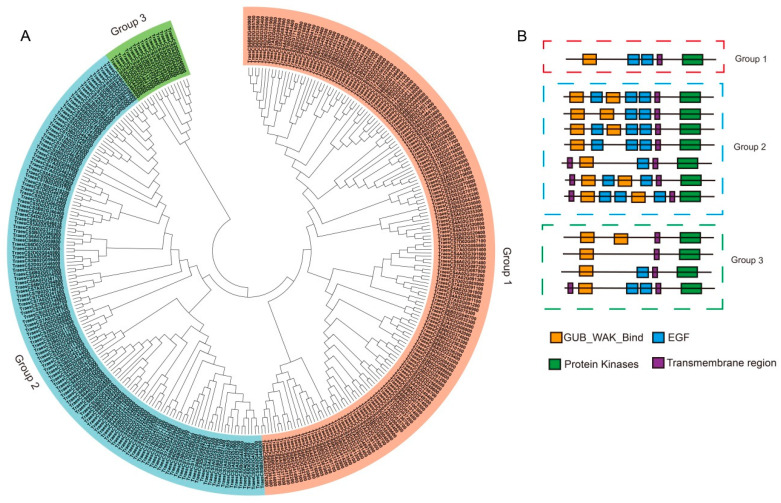
Phylogenetic and structural analysis of TaWAK proteins. (**A**) The phylogenetic tree of wall-associated kinase proteins from *Arabidopsis thaliana* and wheat constructed using the neighbor-joining method. (**B**) Protein structures of TaWAKs. The orange, blue, green, and purple boxes indicate the WAK_GUB domain, epidermal growth factor domain, protein kinase, and transmembrane domain, respectively.

To explore the phylogenetic relationship among the WAKs in wheat, WAK protein sequences were used to construct a phylogenetic tree with the neighbor-joining (NJ) method. All members of the TaWAK family were classified into three groups (Figure 1A): Group 1 was the largest, with 166 TaWAKs; Group 2 was the second largest, with 138 TaWAKs; and Group 3 was the smallest, with 16 TaWAKs. The protein structures were further characterized, and the numbers of the WAK, EGF, and TM domains were determined (Figure 1B). The WAK domains were mostly located in the N-terminal of the proteins, whereas the kinase domains were mostly located in the C-terminal of the WAKs. Group 1 included one protein structure with a WAK-binding domain, two EGF domains, and a typical kinase domain. Group 2 comprised several structures, including varying positions and numbers of EGF structures. Finally, Group 3 included four types of structures with 0–2 EGF domains. All members were predicted to have at least one transmembrane region (Figure 1B).

### 2.2. Chromosomal Location and Duplication of TaWAK Genes in Wheat

**Figure 2:**
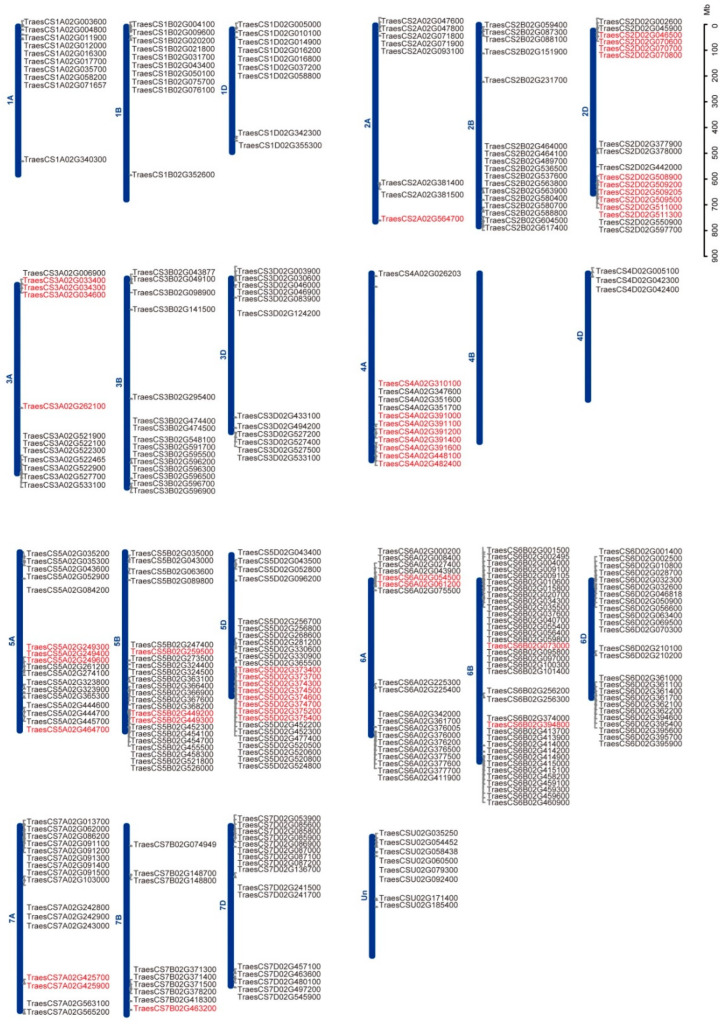
Chromosomal location of the *TaWAK* genes. The distribution of *TaWAKs* on each wheat chromosome with a scale bar is displayed in megabase (Mb). The 45 *TaWAKs* in hcmQTLs are in red.

The chromosomal locations of *TaWAKs* were identified and mapped onto the corresponding wheat chromosomes to determine their genomic distribution (Figure 2). Only eight predicted *TaWAK* genes were marked on the scaffold; others were distributed on all 21 chromosomes except 4B, and some were clustered at the ends of chromosomes. Among them, 94, 110, and 108 *TaWAKs* were identified in the A, B, and D subgenomes, respectively. The numbers of *TaWAKs* in each chromosome varied from a minimum of 3 in chromosome 4D to a maximum of 36 in chromosome 6B. Chromosomes 5 and 6 were the most abundant in *TaWAKs* with 66 and 80 members, respectively. Among the numerous QTLs reported to improve FHB resistance in wheat, 77 high-confidence mQTLs (hcmQTLs) have been identified in wheat [32]. We further investigated the chromosomal locations of both *TaWAKs* and hcmQTLs to explore the potential role of *TaWAKs* in FHB resistance. In total, 45 *TaWAKs* were present in 17 hcmQTLs (Table 1), which provided valuable information that can aid in the discovery of new genes related to improving FHB resistance in wheat.

**Figure 3:**
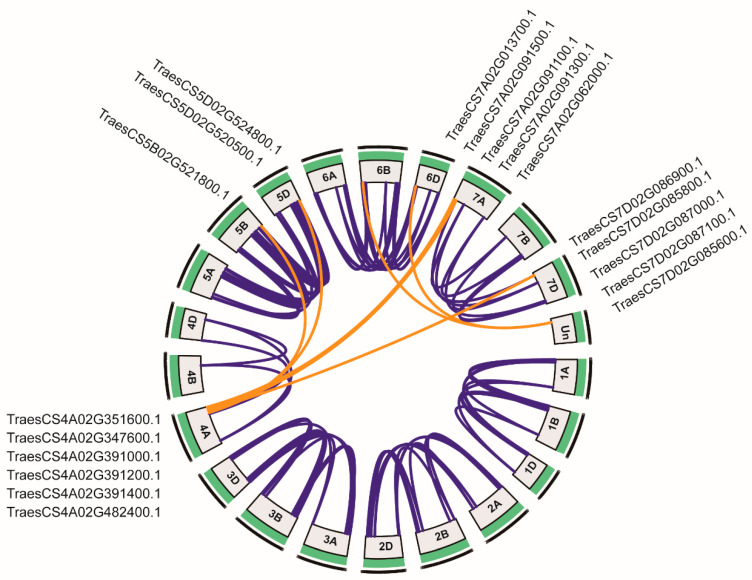
Segmental duplication of *TaWAK* genes. Blue lines denote the segmental duplication of *TaWAK* gene pairs between homologous chromosomes, and the orange lines denote the segmental duplication of *TaWAK* gene pairs between nonhomologous chromosomes.

Segmental and tandem duplications are important factors in gene family expansion. A total of 207 *TaWAKs* participated in duplication events (Figure 3), which indicated that the expansion of the *TaWAK* gene family in wheat could chiefly be attributed to whole-genome duplication or segmental duplication within genomes. A total of 176 pairs of *TaWAK* genes were identified to have undergone segmental duplication, with 21, 12, 27, 18, 38, 45, and 15 pairs located on chromosomes 1, 2, 3, 4, 5, 6, and 7, respectively (Figure 3). As per previous research [33], a tandem duplication event has been defined as the presence of two or more genes in a 200 kb-long segment. Fifty-four tandem duplication events were present on all chromosomes except 1B and 4B (Figure 2 and Figure 3). Six genes on chromosome 4A (*TraesCS4A02G351600*, *TraesCS4A02G347600*, *TraesCS4A02G391000*, *TraesCS4A02G391200*, *TraesCS4A02G391400*, and *TraesCS4A02G482400*) were paired with *WAKs* on the nonhomologous chromosomes 5B, 5D, 7A, and 7D and an unidentified chromosome. These results suggest the involvement of tandem and segmental duplication events in the expansion of the *TaWAK* gene family.

### 2.3. Synteny Analysis of WAKs in Wheat and Other Plants

**Figure 4:**
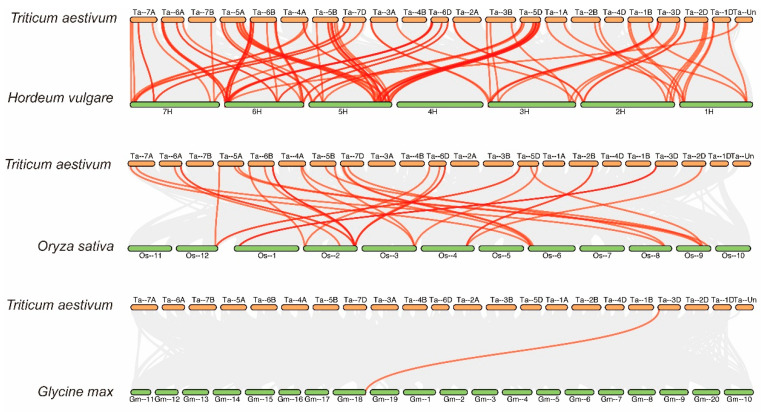
Syntenic relationships of *TaWAK* genes among *Hordeum vulgare*, *Oryza sativa,* and *Glycine max*. The gray lines in the background represent the collinear blocks within *Triticum aestivum* and other plant genomes. The red lines highlight the syntenic *WAK* gene pairs.

To further investigate the syntenic relationship of wheat *TaWAK* genes with other plants, such as barley (*Hordeum vulgare*), rice (*Oryza sativa*), and soybean (*Glycine max*), three comparative syntenic maps were constructed using the Multiple Collinearity Scan toolkit (Figure 4). A total of 101 and 34 orthologous gene pairs were identified between *TaWAKs* and other *WAK* genes in *H. vulgare* and *O. sativa*, respectively (Table S2). Some *TaWAK* family members were found to be associated with more than one syntenic gene pair between wheat and *H. vulgare* as well as between wheat and *O. sativa*; for instance, *HORVU.MOREX.r3.2HG0194210* was associated with *TraesCS2D02G442000* and *TraesCS2B02G464000*, and *TraesCS6D02G361000* was associated with *Os02g0811200* and *Os02g0807200*. However, in the dicotyledonous species *G. max*, only one orthologous gene pair was present between *TaWAKs* and *WAK* genes, which suggests the closer syntenic relationship of *WAK* genes in monocots than in dicots.

### 2.4. Expression Profile Analysis of TaWAK Genes in Various Tissues

**Figure 5:**
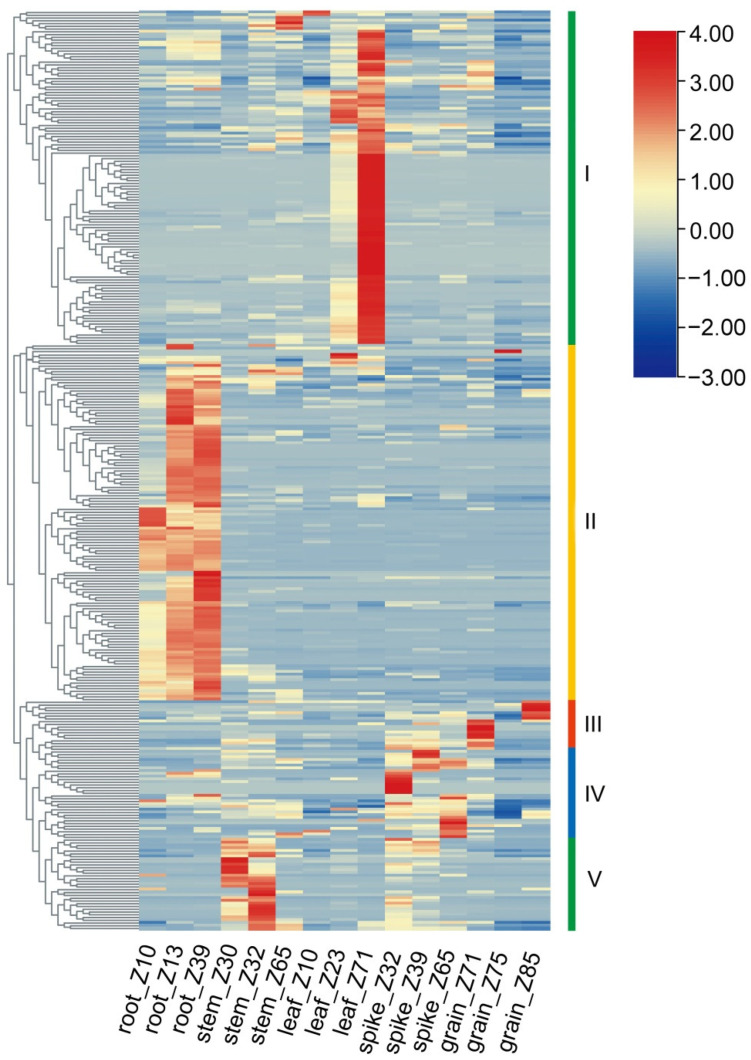
Expression analyses of *TaWAK* genes in five different tissues at three different developmental stages. The *TaWAK* genes were divided into five clusters with different expression patterns.

To understand the spatial and temporal expression patterns of *TaWAK* genes in wheat, transcriptomic data from different developmental stages of the root, stem, leaf, spike, and grain of the cultivar Chinese Spring were analyzed using the Zadoks scale from public data [34]. The expression levels of 307 *TaWAKs* in the five abovementioned tissues were determined and used to construct a heat map (Figure 5). The results revealed that different *TaWAK* genes exhibited differential expression profiles in different tissues and that they could be roughly clustered into five groups according to their specific expression. The expressions of 113 genes in Cluster 1 in the leaf, especially at the Z71 time, exceeded those in other tissues, whereas the expressions of 116 genes in Cluster 2 in the root exceeded those in the other tissues. The numbers of *TaWAKs* highly expressed in Cluster 3, Cluster 4, and Cluster 5 were 14, 20, and 44, respectively.

### 2.5. Expression Profiles of TaWAK Genes in Response to Fusarium Graminearum

**Figure 6:**
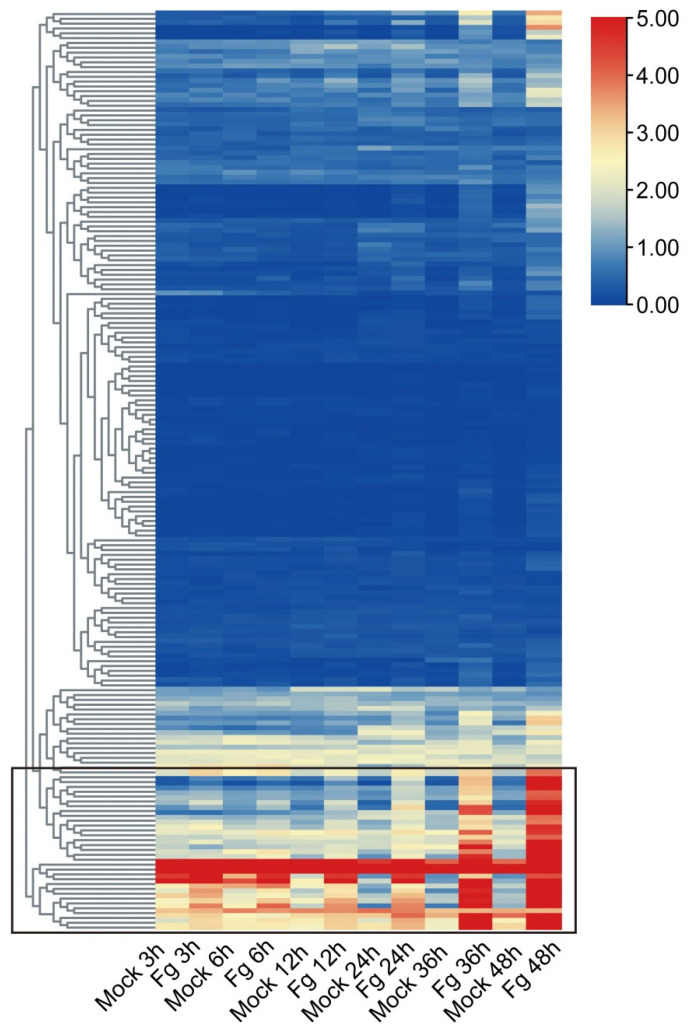
Expression analysis of *TaWAK* genes in response to *Fg* infection. The red color indicates high expression and the blue color indicates low expression. Thirty *TaWAKs* that were upregulated after *Fg* treatment were clustered into a group.

Previous studies have established the significant roles of plant *WAK* genes in the defense against pathogens [5]. To further investigate the putative role of *TaWAKs* in fungal disease resistance, the expression of *TaWAKs* was analyzed using the public RNA-seq data of the wheat spike after inoculation with *Fg* for 3, 6, 12, 24, 36, and 48 h from the WheatGene database. The heatmap showed that although most of the *TaWAKs* either did not change or changed slightly (<2 fold and FPKM < 1) at a series of times after the *Fg* treatment, 30 *TaWAKs* were differentially upregulated at least at one time point (Figure 6). For the upregulated *TaWAKs*, 16 were highly inoculated after 36 and 48 h of *Fg* treatment, whereas 14 were inoculated once after 3 h of *Fg* treatment. These results signify that the 30 *Fg*-responded *TaWAKs* are likely to be involved in wheat FHB resistance.

**Figure 7:**
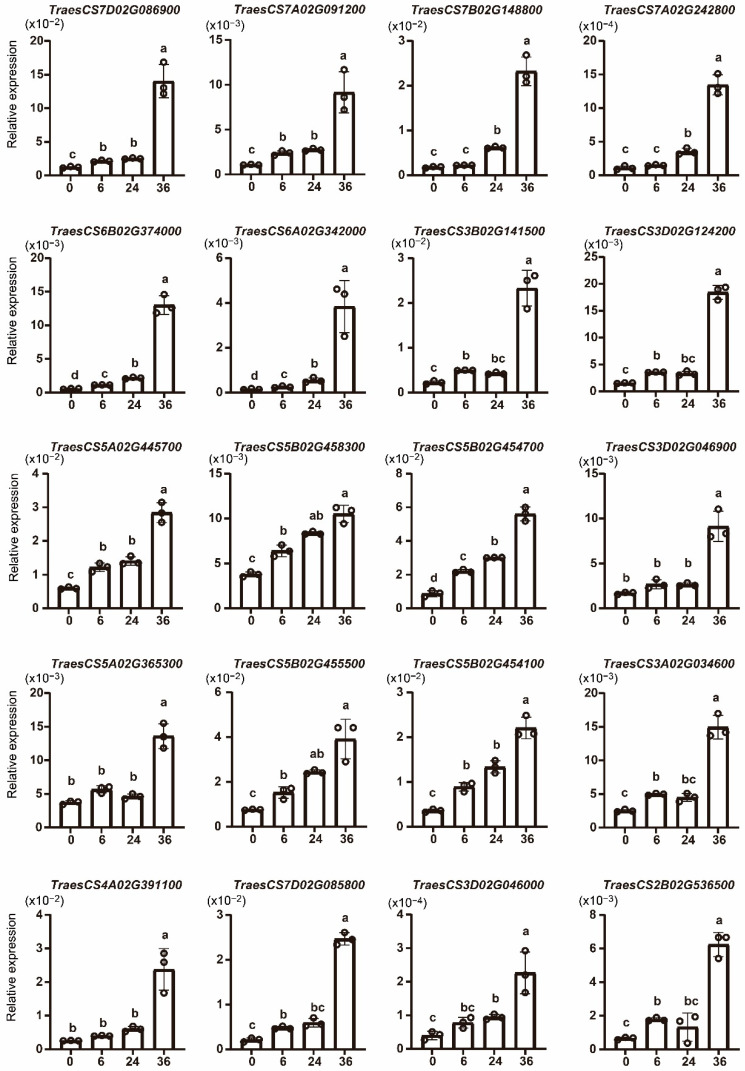
Quantitative real-time PCR analysis of selected *TaWAK* genes in response to 0, 6, 24, and 36 h of *Fg* infection to verify the RNA-seq data. The wheat *tubulin* gene was used as the internal control. The Student’s *t*-test was used to compare the significant differences. Error bars show the standard deviation. Data are mean ± SD (*n* = 3).

To verify the expression pattern of *TaWAKs* after *Fg* infection, 20 of the 30 upregulated *TaWAKs* were evaluated in the wheat spike after 6, 24, and 36 h of *Fg* infection using qRT-PCR. The findings showed that all 20 selected *TaWAKs* were significantly upregulated after 36 h of *Fg* treatment (Figure 7). For the 6 and 24 h *Fg* treatments, most of the *TaWAKs* were slightly but significantly incubated compared with the 0 h treatment. The *TraesCS6A02G342000* gene demonstrated the highest relative expression at 36 h of *Fg* treatment compared with the rest of the tested genes. These results are in agreement with the RNA-seq data and indicate that these *TaWAKs* may be involved in the response to *Fg* infection.

### 2.6. Pectin- and Chitin-Induced TaWAK Genes

**Figure 8:**
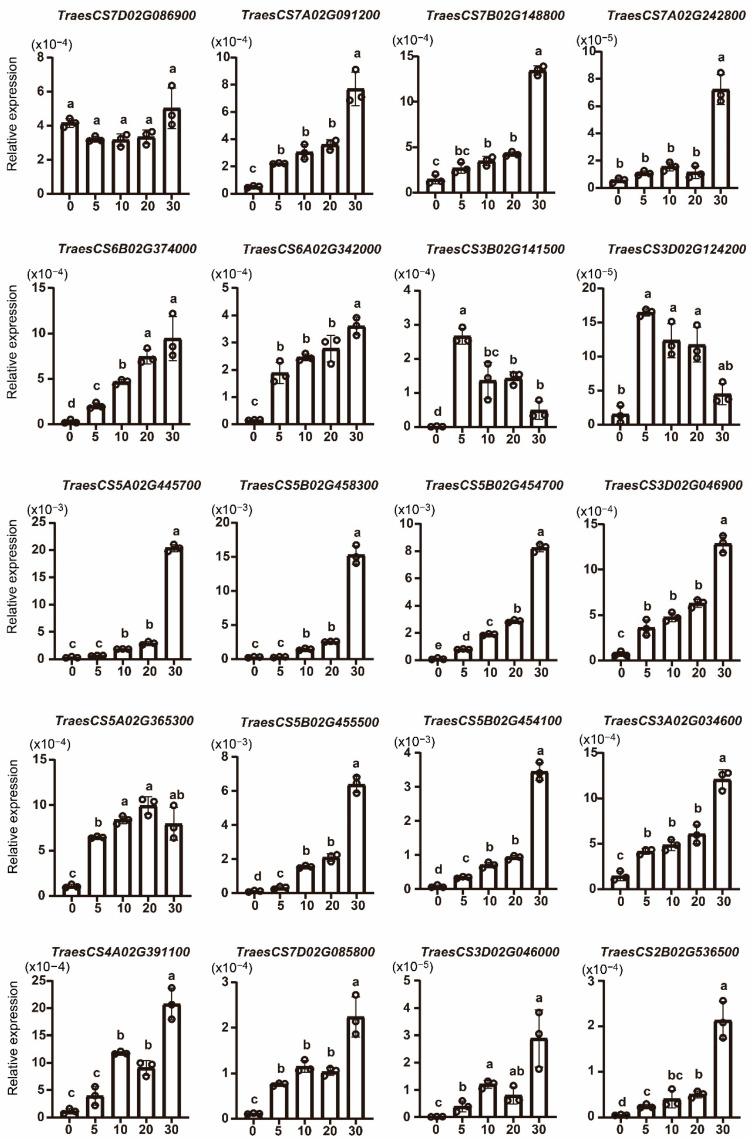
Expression analysis of *TaWAK* genes in wheat after treatment with exogenous pectin for 0, 5, 10, 20, and 30 min. The wheat *tubulin* gene was used as the internal control. The Student’s *t*-test was performed to compare the significant differences. Error bars show the standard deviation. Data are mean ± SD (*n* = 3).

**Figure 9:**
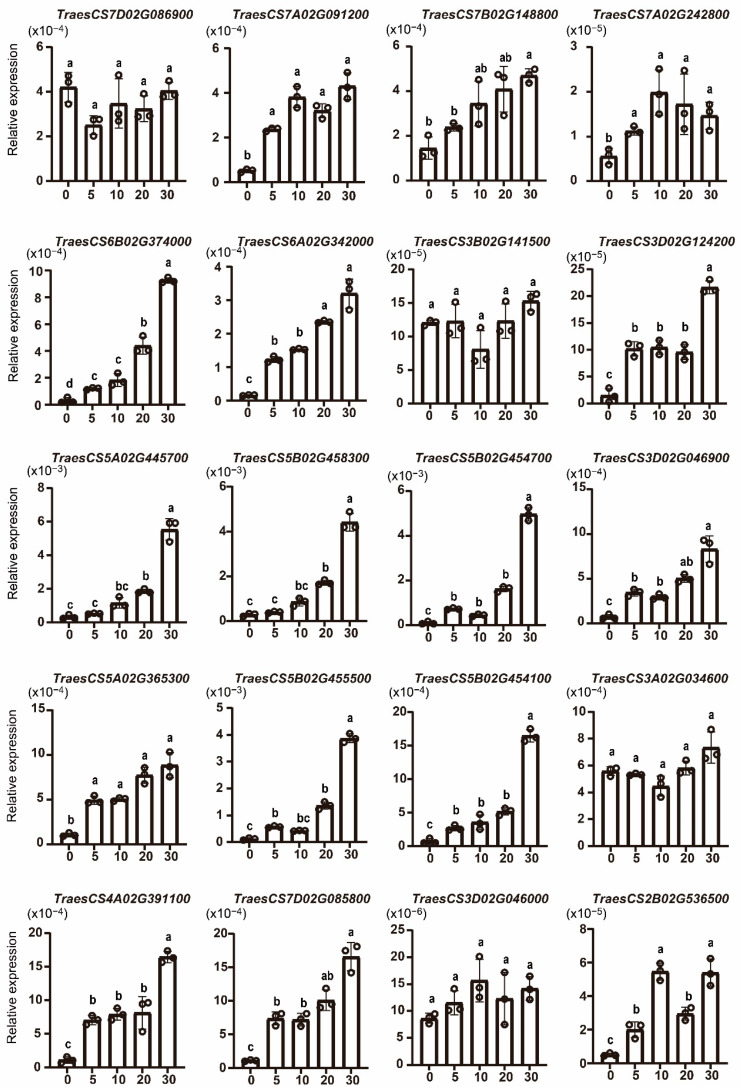
Expression analysis of *TaWAK* genes in wheat after treatment with exogenous chitin for 0, 5, 10, 20, and 30 min. The wheat *tubulin* gene was used as the internal control, and a Student’s *t*-test was used to compare the significant differences. Error bars show the standard deviation. Data are mean ± SD (*n* = 3).

Pectin and chitin act as elicitors that trigger the plant’s defense response. To investigate the response of *TaWAKs* to exogenous pectin and chitin stimuli, we analyzed the transcriptional profiles of *TaWAKs* in wheat spikes treated with either pectin (100 µg/mL) or chitin (100 µg/mL), or mock solution for 0, 5, 10, 20, and 30 min. Most of the *TaWAKs* were elevated over time and reached their highest expression level after 30 min of exogenous pectin treatment. Only *TraesCS3B02G141500* and *TraesCS3D02G124200* were induced at 5 min and then decreased gradually (Figure 8). In the exogenous chitin treatment, 12 *TaWAKs* were elevated over time and reached their highest expression level after 30 min (Figure 9). *TraesCS7D02G086900* was not induced by pectin or chitin treatment. *TraesCS3B02G141500*, *TraesCS3A02G034600*, and *TraesCS3D02G046000* transcript levels were significantly elevated by exogenous pectin treatment but not by chitin treatment compared with the mock treatment. Collectively, these results suggest that the *TaWAK* family may contribute to pectin- and chitin-induced defense pathways in wheat.

### 2.7. Subcellular Localization of TaWAK Proteins

**Figure 10:**
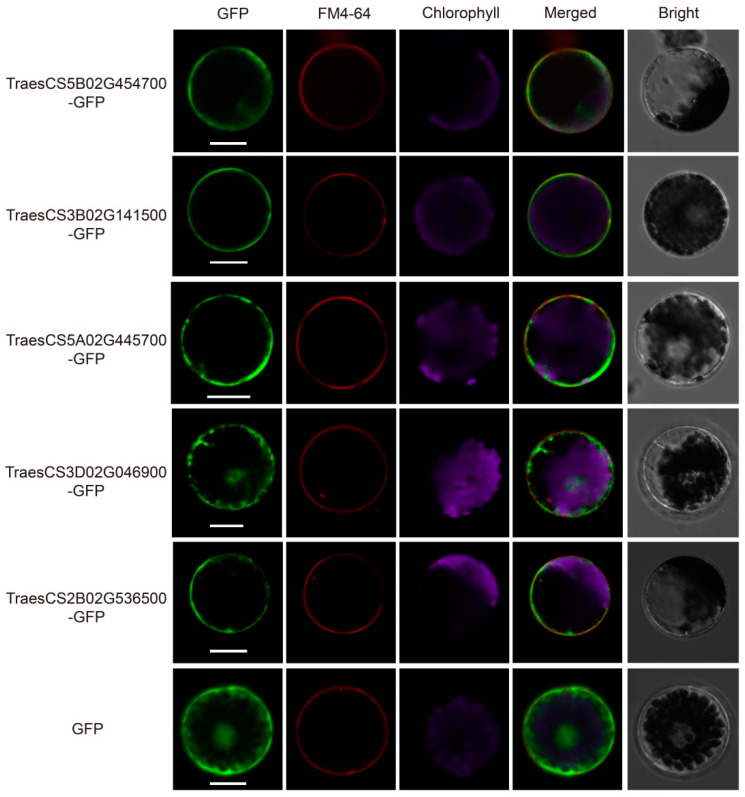
Subcellular localization of TaWAK proteins in wheat protoplasts. Plasmids labeled with GFP were separately transferred into the wheat protoplast, and the amphiphilic styryl dye FM4-64 was used as a plasma membrane marker. Scale bar = 20 μm.

To investigate the subcellular localization of TaWAKs in wheat, Five *TaWAK* gene sequences from the abovementioned qRT-PCR analysis were fused with the *green fluorescent protein* (*GFP*) reporter gene in the presence of the maize ubiquitin promoter. The resulting fusion proteins and the control ubi:GFP were transiently expressed in the wheat protoplasts. The amphiphilic styryl dye FM4-64 was used as the plasma membrane marker owing to its ability to immediately stain the plasma membrane after application in plants [35]. Confocal microscopy revealed that the GFP fluorescence of four proteins, TraesCS5B02G454700, TraesCS3B02G141500, TraesCS5A02G445700, and TraesCS2B02G536500, was distributed in the plasma membrane of wheat protoplasts, just as FM4-64, whereas TraesCS3D02G046900 was expressed in all parts of the protoplasts, just as the GFP control (Figure 10). These results denote that TaWAKs have different subcellular localizations and varying functions in wheat.

## 3. Discussion

WAKs are important RLKs that typically comprise a putative extracellular domain, a hydrophobic TM region, and a cytoplasmic Ser/Thr kinase domain. It has been shown that WAKs function in signal transduction between the extracellular matrix and the cytoplasm and play pertinent roles in plant growth and development [26]. WAKs have recently gained attention as key factors in plant defense against pathogens [5]. Characterizing the *WAK* gene family will help identify many new candidate genes that may contribute to disease resistance. Presently, *WAK* gene families have been identified and characterized in diverse plant species, including 26 members in Arabidopsis [3], 125 in rice [27], 91 in barley [28], 27 in walnuts [29], 29 in tomato [25], 29 in potato [36], and 29 in cotton [26]. Although several wheat WAKs have been reported, their genome-wide characterization in the complicated wheat hexaploid genome is lacking.

In this study, 320 *TaWAK* genes containing the typical WAK_GUB, TM, and protein kinase domains were identified and characterized based on a high-quality version of the wheat genome assembled and annotated by IWGSC [30]. These genes were classified into three groups based on phylogenetic analysis. For the four typical domains, the protein structures were relatively conserved in each group, with only one similar structure for 166 TaWAK members in Group 1. The relative MW of the proteins was in the range of 65.4–119.2 kDa, and the range of the theoretical isoelectric point was 5.09–9.24, thus indicating a great functional diversity in TaWAK proteins. Chromosomal mapping of the *TaWAK* genes revealed that the 320 *TaWAKs* were almost uniformly distributed on three subgenomes, with 94, 110, and 108 genes in the A, B, and D subgenomes but unevenly distributed on each chromosome. The gene number on each chromosome varied from 0 in chromosome 4B to 36 in chromosome 6B. The number of *WAK* genes in wheat is quite high compared with rice and barley, and approximately >10 times that in dicotyledonous plants such as Arabidopsis and potato [3,27,28,36]. This variation could be attributed to the fact that wheat has a large genome with two whole-genome duplications, which implies more occurrences of *WAK* gene duplication events during domestication in monocotyledons than in dicotyledons. In fact, 297 of the 320 *TaWAKs* participated in duplication events. Further analysis revealed that 176 pairs of *TaWAK* genes were segmental duplications and that 54 tandem duplication events were concentrated on all chromosomes, except for chromosomes 1B and 4B. Moreover, *WAK* gene duplication events have been reported in other plant species such as cotton, rice, *J. regia,* and *J. mandshurica*. Thus, whole-genome and segmental duplications are among the common causes of *WAK* gene family expansion in different species [26,27,29]. The syntenic relationship revealed the presence of 101 and 34 orthologous gene pairs between wheat and *H. vulgare* and *O. sativa,* respectively, and only one orthologous gene pair between wheat and *G. max*. This finding suggests that *TaWAK* genes in wheat originated from a common ancestor during the evolutionary process in monocotyledonous plants but are distantly related to those in dicotyledonous species.

*WAK* genes exhibit selective and differential expression patterns in several plant species. For instance, *GhWAKs* have been observed to be expressed in a tissue-specific manner in cotton, with some highly expressed in young roots and some in a flower-specific manner [26]. *TaWAKs* have also been found to be expressed differently in different tissues and developmental stages, with five tissue-specific highly expressed pattern groups. For example, *TraesCS7D02G086900*, *TraesCS7B02G148800*, and *TraesCS6B02G374000* genes were chiefly expressed in the leaf, root, and spike, respectively. The variations in the expression levels of *TaWAKs* indicate that these genes may be involved in the control of diverse biological processes related to the specific expression patterns shown in the corresponding tissues.

Several independent studies have established that WAK family proteins constitute a central pillar of plant cells that monitor and interact with their extracellular environments at both transcriptional and post-transcriptional levels. Moreover, they are important in the defense against pathogens and contribute to plant immunity [5,13,15,18,37,38,39]. Most of these immune-related *WAK* genes are highly induced upon infection by a pathogen. For example, *GhWAK7A* showed enhanced expression at 1 d after inoculation with *V. dahliae* [39], the expression of *OsWAK25* was upregulated upon *M. oryzae* infection [6], and the *TaWAK7D* transcript level was increased after inoculation with *R. cerealis* [20]. FHB, mainly caused by *Fg*, is among the most widespread and devastating crop diseases and can exert damaging effects on wheat yield and quality. To identify new *TaWAK* genes essential for FHB resistance, the expression pattern of *TaWAKs* was analyzed. Although most of the *TaWAKs* did not change or changed slightly at a series of time points after *Fg* treatment, 30 *TaWAKs* were differentially upregulated. Twenty *TaWAKs* were further evaluated in the spike after 6, 24, and 36 h of *Fg* infection by qRT-PCR, indicating that they may be involved in the response to *Fg* infection.

For the genes that were not induced in this study, the possibility of their participation in FHB resistance cannot be eliminated. For example, *TaWAK2A-800* reportedly participates positively in the resistance responses to FHB; however, it was significantly induced upon *Fg* inoculation in the FHB-resistant wheat cultivar, Sumai 3 [24] but not in this study. This difference may result from the expression analysis in different varieties or the conditions of *Fg* treatment. To date, numerous QTLs distributed over all 21 wheat chromosomes have been reported, and 77 high-confidence mQTLs (hcmQTLs) have been recently selected [32]. In this study, 45 *TaWAKs* in 17 hcmQTLs were identified, thereby providing valuable information for discovering new *TaWAK* genes related to improving FHB resistance in wheat.

Pectin and chitin are typical PAMPs on the fungal cell wall, which act as important elicitors in several plant–pathogen interactions [8,40]. According to previous research, pectin and chitin can trigger the expression of *WAK* genes. For example, *TaWAK7D* transcript levels were elevated after pectin and chitin treatments [20], *TaWAK2A-800* was significantly induced by chitin treatment [24], and chitin triggered the expression of the rice *OsWAK91/92/14* genes [14]. In this study, 19 of the 20 selected *TaWAKs* were elevated after exogenous pectin treatment, and 12 of the 20 *TaWAKs* were induced after exogenous chitin treatment, thus demonstrating that the *TaWAK* family may contribute to the pectin- and chitin-induced defense pathway in wheat. Many disease-resistant WAKs, including CsWAKL08 [41], OsWAK1 [4], ZmHtn1 [38], TaWAK-6D [21], and TaWAK7D, have been found to be localized in the plasma membrane [20]. We selected five TaWAKs to investigate their subcellular localization and observed that four were distributed in the plasma membrane, whereas the other one was expressed in both the plasma membrane and the nucleus. These findings suggest that the plasma membrane distribution of these TaWAKs may be responsible for their immune receptor roles, but the roles of these proteins in nuclear localization require further clarification.

## 4. Materials and Methods

### 4.1. Identification of the WAK Family in Wheat

The latest wheat reference genome (IWGSC) and hidden Markov model (HMM) profile (PF08488) of the WAK family were retrieved from the Ensembl Plants website (http://plants.ensembl.org/index.html accessed on 9 August 2021) and protein family database (Pfam) (http://pfam.sanger.ac.uk/ accessed on 9 August 2021) website. The AtWAK protein sequences of *Arabidopsis thaliana* obtained from the Arabidopsis Information Resource database (https://www.arabidopsis.org/ accessed on 9 August 2021) as previously described [3] were used as queries to identify putative WAK genes in bread wheat in a local database using BLASTp. The WAK HMM profile was employed for functional annotation filters using HMMER software (version 3.0). Subsequently, all candidate protein sequences were further verified for the existence of WAK_GUB, TM, EGF, and protein kinase domains using the Pfam (http://pfam.xfam.org/ accessed on 22 August 2021) and SMART (http://smart.embl-heidelberg.de/ accessed on 22 August 2021) websites. Finally, the proteins that coexisted with the WAK_GUB, TM, and protein kinase domains were considered TaWAKs. MW and pI were predicted using the ExPasy website (https://www.expasy.org/ accessed on 26 August 2021).

### 4.2. Phylogenetic Analysis

Multiple sequence alignment of the full-length amino acid sequences of TaWAKs was initially performed using MUSCLE (http://www.ebi.ac.uk/Tools/msa/muscle/ accessed on 6 September 2021). An unrooted NJ phylogenetic tree was constructed using MEGA 6.0 with the parameters as follows: pairwise deletion, p-distance model, and 1000 bootstrap replications [42].

### 4.3. Chromosomal Distribution, Gene Duplication, and Synteny Analysis

The chromosomal distribution of each *TaWAK* gene was obtained from the wheat genome database published in IWGSC [30]. The physical map of the *TaWAKs* on the chromosomes of wheat was visualized using TBtools software (version v.1.098696) [43]. Tandem and segmental duplications in the TaWAK family were investigated using a Multiple Collinearity Scan toolkit (MCScanX) [44]. The genome information of *H. vulgare*, *O. sativa,* and *G. max* was downloaded from the Ensemble website. The syntenic relationships of the WAK family between bread wheat and *H. vulgare*, *O. sativa,* and *G. max* were analyzed using MCScanX. Segmental and tandem duplication relationships were virtualized using the Advanced Cicros function of TBtools software (version v.1.098696).

### 4.4. Expression Profiling of TaWAK Genes

RNA-seq data from root, stem, leaf, spike, and grain, each with three developmental stages, were retrieved from the Wheat Expression Browser (http://www.wheat-expression.com accessed on 28 September 2021) [34]. RNA-seq data from the wheat spike after *Fg* inoculation for 3, 6, 12, 24, 36, and 48 h were retrieved from the WheatGene database (http://wheatgene.agrinome.org/gene-expression-analysis accessed on 28 September 2021). The heat map was generated using TBtools (version v.1.098696).

### 4.5. RNA Isolation and qRT-PCR

The seeds of the wheat genotype Fiedler were grown in plastic pots in growth chambers in Nanjing at a relative humidity of 50% and a 16 h photoperiod under 22 °C (light) and 18 °C (dark) conditions. The spike at the heading stage was infected by *Fg* (Fg1312) for 0, 6, 24, and 36 h. The inoculated spike was covered with plastic bags to maintain humidity and promote *Fg* infection. The spike at the heading stage was treated with pectin (100 µg/mL) or chitin (100 µg/mL) for 0, 5, 10, 20, and 30 min.

Total RNA was extracted using TRIzol reagent (Invitrogen, Carlsbad, CA, USA). The quality and quantity of each RNA extract were determined with agarose gel electrophoresis and Nanodrop 2000 (Thermo Fisher Scientific, Carlsbad, CA, USA). Subsequently, the RNA was purified and reverse-transcribed into cDNA using the FastQuant RT Kit (Takara, RR047A, Dalian, China). qRT-PCR was performed using the Roche Thermal Cycler 96 with the TB Green^®^ Premix Ex Taq™ II reagent (Takara, RR820B, Dalian, China). A total of 10 µL reaction solution, including 5 µL of TB Green Enzyme, 3.2 µL of water, and 0.6 µL of primer, was used for qRT-PCR. The PCR procedure was performed as described previously [45], which started at 95 °C for 30 s, followed by 45 cycles of 95 °C for 5 s, 60 °C for 20 s, and 72 °C for 10 s. Melting curve analysis included 95 °C for 10 s, 65 °C for 15 s, and heating to 95 °C at a rate of 0.1 °C/s with continuous readings of fluorescence for each amplification. The wheat *tubulin* gene [46] was used as the normalization reference gene for the gene expression analysis. Table S3 shows the gene-specific primers used for qRT-PCR. All reactions were run in triplicate, technical replicates for each sample, and three biological replicates were performed.

### 4.6. Subcellular Localization of TaWAKs

The coding region of *TaWAKs* lacking the stop codon was amplified using gene-specific primers (Table S3). The amplified fragment was digested with restriction enzyme Kpn I and subcloned in-frame at the 5′-terminus of the GFP coding region in the pCAMBIA1300 vector with the maize ubiquitin promoter. The Ubi:TaWAK-GFP fusion construct or the ubi:GFP control construct was individually introduced into wheat protoplasts. GFP signals were detected and photographed using a confocal laser scanning microscope after incubation at 21 °C for 16 h (Zeiss LSM 980, Oberkochen, Germany).

### 4.7. Statistical Analysis

All experiments were conducted at least three times with technical and biological replicates. Data were analyzed using Microsoft Excel. A two-tailed Student’s t-test was used to compare the significant differences. Values of *p* < 0.05 were considered statistically significant.

## 5. Conclusions

WAKs are important RLKs and play key roles in plant defense against pathogens. FHB is one of the most widespread and devastating crop diseases, causing wheat yield reduction and quality deterioration. We identified and characterized 320 *TaWAK* family members on all the chromosomes except 4B in bread wheat; these members could be categorized into three phylogenetic groups. Duplication and synteny analysis yielded valuable information on the evolutionary characteristics of *TaWAK* genes. Thirty *TaWAKs* were incubated after *Fg* treatment and most of them could contribute to pectin- and chitin-induced defense pathways. Additionally, 45 *TaWAKs* in 17 FHB resistance-related hcmQTLs were identified in this study. These results provide valuable information that can be used for discovering new *TaWAK* genes to improve FHB resistance in wheat.

## Figures and Tables

**Table 1:** List of *TaWAKs* in FHB-related hcmQTLs in wheat.

Gene Name	hcmQTL	Interval (Mb)	Chr.
TraesCS2A02G564700	hcmQTL-13	763–771	2A
TraesCS2D02G046500	hcmQTL-18	17–23	2D
TraesCS2D02G070600	hcmQTL-19	26–36	2D
TraesCS2D02G070700	hcmQTL-19	26–36	2D
TraesCS2D02G070800	hcmQTL-19	26–36	2D
TraesCS2D02G508900	hcmQTL-21	592–608	2D
TraesCS2D02G509200	hcmQTL-21	592–608	2D
TraesCS2D02G509205	hcmQTL-21	592–608	2D
TraesCS2D02G509500	hcmQTL-21	592–608	2D
TraesCS2D02G511000	hcmQTL-21	592–608	2D
TraesCS2D02G511300	hcmQTL-21	592–608	2D
TraesCS3A02G033400	hcmQTL-23	15–21	3A
TraesCS3A02G034300	hcmQTL-23	15–21	3A
TraesCS3A02G034600	hcmQTL-23	15–21	3A
TraesCS3A02G262100	hcmQTL-28	466–485	3A
TraesCS4A02G310100	hcmQTL-34	594–613	4A
TraesCS4A02G391000	hcmQTL-35	660–676	4A
TraesCS4A02G391100	hcmQTL-35	660–676	4A
TraesCS4A02G391200	hcmQTL-35	660–676	4A
TraesCS4A02G391400	hcmQTL-35	660–676	4A
TraesCS4A02G391600	hcmQTL-35	660–676	4A
TraesCS4A02G448100	hcmQTL-36	712–720	4A
TraesCS4A02G482400	hcmQTL-37	728–743	4A
TraesCS5A02G249300	hcmQTL-46	464–472	5A
TraesCS5A02G249400	hcmQTL-46	464–472	5A
TraesCS5A02G249600	hcmQTL-46	464–472	5A
TraesCS5A02G464700	hcmQTL-47	644–662	5A
TraesCS5B02G259500	hcmQTL-50	438–448	5B
TraesCS5B02G449200	hcmQTL-53	610–623	5B
TraesCS5B02G449300	hcmQTL-53	610–623	5B
TraesCS5D02G373400	hcmQTL-55	444–454	5D
TraesCS5D02G373700	hcmQTL-55	444–454	5D
TraesCS5D02G374300	hcmQTL-55	444–454	5D
TraesCS5D02G374500	hcmQTL-55	444–454	5D
TraesCS5D02G374600	hcmQTL-55	444–454	5D
TraesCS5D02G374700	hcmQTL-55	444–454	5D
TraesCS5D02G375200	hcmQTL-55	444–454	5D
TraesCS5D02G375400	hcmQTL-55	444–454	5D
TraesCS6A02G054500	hcmQTL-58	26–35	6A
TraesCS6A02G061200	hcmQTL-58	26–35	6A
TraesCS6B02G073000	hcmQTL-61	47–65	6B
TraesCS6B02G394800	hcmQTL-65	662–682	6B
TraesCS7A02G425700	hcmQTL-68	611–629	7A
TraesCS7A02G425900	hcmQTL-68	611–629	7A
TraesCS7B02G463200	hcmQTL-74	709–728	7B

## Data Availability

All data used or analyzed in this study are included in this published article and its supplementary materials.

## Supplementary Materials

The following supporting information can be downloaded at https://www.mdpi.com/article/10.3390/ijms23137157/s1.
