# Supplementary material for: Characterization of the WAK Gene Family Reveals Genes for FHB Resistance in Bread Wheat (Triticum aestivum L.)
Source: Int J Mol Sci. 2022 Jun 28;23(13):7157. doi: 10.3390/ijms23137157 (PMC9266398; doi:10.3390/ijms23137157)
Supplement: Supplementary file 1 [file ijms-23-07157-s001.zip › Table S1.pdf]

Table S1. The detailed information of 320 *TaWAK* genes.

Gene ID	Chr	Chromosomal location		PI	MW
TraesCS6B02G414200	6B	687305705	687308164	6.14	85041.98
TraesCS6B02G020700	6B	12653307	12656688	5.63	103327.2
TraesCS6B02G459100	6B	713755448	713760135	5.98	106273.49
TraesCS3A02G034600	3A	19471388	19506635	7.33	76248.78
TraesCS1A02G004800	1A	2934676	2938176	6.69	71344.85
TraesCS1D02G037200	1D	17627901	17630762	5.77	82090.44
TraesCS2D02G597700	2D	650076890	650079892	6.45	79261.9
TraesCS6D02G046818	6D	21077215	21079777	8.65	87037.1
TraesCS5B02G455500	5B	629895667	629898862	6.37	78049.81
TraesCS6A02G376000	6A	598064855	598067329	8.16	82571.83
TraesCS4D02G042400	4D	19774280	19784471	6.19	77950.18
TraesCS2B02G563800	2B	755591295	755594459	8.79	77601.44
TraesCS1A02G071657	1A	54015302	54019064	6.42	74069.89
TraesCS7B02G148700	7B	196377969	196380924	5.8	82771.48
TraesCS2B02G489700	2B	687205211	687207726	6.19	82267.07
TraesCS2D02G045900	2D	16762900	16765259	8.26	81076.14
TraesCS7B02G148800	7B	196382223	196385469	5.83	81366.48
TraesCS6B02G009105	6B	5793738	5796725	6.52	68748.04
TraesCS6A02G054500	6A	28410489	28412881	6.98	81064.58
TraesCS7A02G091200	7A	55382439	55385069	5.6	85104.06
TraesCS5D02G268600	5D	371844581	371850931	5.81	82010.75
TraesCSU02G185400	Un	279469323	279473147	5.71	83115.04
TraesCS3D02G030600	3D	11333642	11355705	8.07	71375.23
TraesCS5D02G375200	5D	447631020	447635524	6.42	116522.83
TraesCS7D02G241500	7D	205737331	205745926	5.61	77617.22
TraesCS3B02G043877	3B	22424706	22431800	7.75	104863.4
TraesCS5A02G035200	5A	32705951	32708571	7.62	86500.34
TraesCS6A02G411900	6A	613969591	613976261	5.98	105017.54
TraesCS6A02G075500	6A	45652841	45655794	5.94	82223.48
TraesCS7A02G103000	7A	63125013	63127930	6.66	78932.36
TraesCS7A02G243000	7A	218255715	218259563	6.95	74376.04
TraesCS2D02G511000	2D	602697291	602705377	7.96	81959.89
TraesCS6A02G225300	6A	423521951	423524411	5.61	84010.26
TraesCS6B02G004000	6B	3122693	3127984	6.52	100275.78
TraesCS4A02G391200	4A	668093684	668096362	5.64	79381.15
TraesCSU02G054452	Un	42526297	42529678	7.61	100848.88
TraesCS7D02G136700	7D	87995980	87998917	5.83	82201.78
TraesCS6B02G097000	6B	73162947	73175855	5.77	117670.53
TraesCSU02G079300	Un	71305191	71308194	5.65	81845.2
TraesCS7B02G371400	7B	637478137	637496216	5.97	79002
TraesCS5D02G281200	5D	382487986	382490384	6.63	76801.6
TraesCS2B02G604500	2B	786102579	786105550	7.18	83608.78
TraesCS5B02G063600	5B	71514389	71521162	6.86	76464.42
TraesCS6B02G009100	6B	5790320	5793270	6.1	69523.1
TraesCS2B02G231700	2B	228758409	228761398	6.16	80226.75
TraesCS3D02G003900	3D	1655945	1659437	5.72	82785.31
TraesCS5D02G375400	5D	447704942	447707718	6.57	84779.94
TraesCS4A02G351600	4A	627355663	627358542	5.65	82765.36
TraesCS1D02G010100	1D	5421419	5424921	5.9	83041.99

TraesCS3A02G522465	3A	738502105	738504535	8.2	66216.53
TraesCS5A02G274100	5A	482902191	482904566	7.15	76870.74
TraesCS6B02G037600	6B	22081260	22105393	5.57	101481.11
TraesCS5A02G323900	5A	535528236	535531477	5.86	84271.59
TraesCS3B02G591700	3B	816170822	816173759	6.16	92096.68
TraesCSU02G058438	Un	45771082	45773544	6.03	73359.01
TraesCS6D02G032600	6D	13724219	13735758	5.72	101545.25
TraesCS7D02G085900	7D	52829591	52832227	6	87140.72
TraesCS2B02G617400	2B	793971622	793975519	5.98	78945.72
TraesCS2B02G537600	2B	733062825	733073795	7.29	81115.65
TraesCS6D02G395900	6D	468180987	468185160	5.95	82316.73
TraesCS5B02G366400	5B	545278284	545280859	6.08	83794.6
TraesCS3D02G527200	3D	605704741	605708211	5.85	79445.05
TraesCS7A02G091300	7A	55395188	55398052	5.69	81235.4
TraesCS5D02G256700	5D	362885595	362890154	5.6	81277.67
TraesCS7B02G463200	7B	720131495	720134235	5.87	70497.51
TraesCS5B02G458300	5B	633175437	633179348	7.06	72897.44
TraesCS7D02G087100	7D	53482822	53485447	6.06	85267.09
TraesCS2A02G381500	2A	624965404	624967932	5.33	82083.89
TraesCS6B02G394800	6B	670209284	670213989	5.34	92852.15
TraesCS6B02G460900	6B	714329651	714336717	7.15	83781.52
TraesCS1A02G012000	1A	6899512	6903022	6.37	82880.2
TraesCS1D02G016800	1D	7583590	7587977	5.96	75504.75
TraesCS3B02G596700	3B	819502902	819507260	6.46	103184.7
TraesCS5B02G449300	5B	621476503	621480029	5.76	102157.91
TraesCS3D02G533100	3D	608924067	608931731	5.85	114802.61
TraesCS4A02G351700	4A	627360276	627363268	5.79	83802.28
TraesCS6D02G050900	6D	24375492	24377880	5.84	81917.72
TraesCS6A02G342000	6A	574524921	574530023	6.07	82577.24
TraesCS5D02G052800	5D	50635242	50646756	8.89	83848.67
TraesCS1D02G355300	1D	439927726	439931083	5.75	78183.08
TraesCS4D02G005100	4D	2990063	3000709	8.75	80576.42
TraesCS7A02G091100	7A	55374863	55377493	5.65	86538.78
TraesCS7B02G371300	7B	637351112	637364971	6.49	99688.33
TraesCS1B02G076100	1B	58930827	58933501	5.9	74782.58
TraesCS3B02G548100	3B	783618086	783623334	6.19	118333.87
TraesCS2D02G508900	2D	601602225	601606633	6.09	82858.43
TraesCS6A02G027400	6A	13806565	13809820	6.48	81566.46
TraesCS1D02G058800	1D	38726541	38729640	6.08	74099.66
TraesCS5D02G524800	5D	543608944	543612338	7.75	103231.05
TraesCS3B02G596200	3B	819186643	819192470	5.89	115196.91
TraesCS4A02G391400	4A	668146294	668148962	7.11	83175.04
TraesCS6D02G362100	6D	452839647	452842635	5.99	82613.39
TraesCS5D02G330900	5D	422000098	422003258	6.18	83461.05
TraesCS2B02G464000	2B	657842837	657845275	6.3	80321.79
TraesCS5A02G445700	5A	625732514	625738763	5.67	77846.31
TraesCS6A02G061200	6A	32897729	32900823	6.13	79581.1
TraesCS5B02G324500	5B	508680522	508683749	6.28	84153.5
TraesCS6B02G015800	6B	9281806	9286139	6.68	75642.12
TraesCS5B02G035000	5B	38157666	38160207	5.46	81123.5
TraesCS6D02G361100	6D	452472272	452474723	8.32	81787.99

TraesCS2B02G087300	2B	49329177	49334563	7.14	78919.25
TraesCS5B02G043000	5B	47589242	47593579	8.79	84497.22
TraesCS5D02G520500	5D	541934412	541937271	6.05	83238.8
TraesCS2A02G071900	2A	31970535	31978848	8.06	78931.23
TraesCS3B02G141500	3B	128363963	128370419	6.29	69505.32
TraesCS5D02G096200	5D	106519841	106525422	5.72	78332.24
TraesCS5A02G249300	5A	464158592	464170977	5.11	83909.39
TraesCS6D02G395600	6D	468095605	468100014	6.68	82561.11
TraesCS2B02G151900	2B	118629530	118632855	6.61	81666.09
TraesCS6D02G063400	6D	29768995	29772072	5.87	79168.52
TraesCS7D02G463600	7D	578790440	578793519	5.48	84146.01
TraesCS2D02G511300	2D	602739787	602775064	6.23	81490.59
TraesCS7A02G242900	7A	218244096	218247173	5.65	82955.81
TraesCS5B02G367600	5B	546093997	546097128	6.21	83475.26
TraesCS7D02G545900	7D	632857507	632860030	5.9	81236.39
TraesCS1A02G003600	1A	2229188	2236819	6.36	103083.87
TraesCS2B02G563900	2B	755626836	755629791	6.33	79434.04
TraesCS1B02G009600	1B	4785129	4791410	6.18	83317.18
TraesCS6D02G361700	6D	452585736	452588189	6.17	84839.64
TraesCS5A02G043600	5A	39916174	39930209	9.24	80528.05
TraesCS1B02G352600	1B	583398724	583406067	7.69	83607.31
TraesCS7A02G565200	7A	734686118	734688652	6.43	81176.55
TraesCS2B02G588800	2B	775357048	775360836	5.77	114038.5
TraesCSU02G171400	Un	252681686	252684252	6.81	69625.32
TraesCS7D02G497200	7D	603792646	603796040	6.43	79367.34
TraesCS7D02G085800	7D	52821521	52823989	5.59	79854.09
TraesCS6B02G413700	6B	687096577	687099050	6.31	81586.36
TraesCS3D02G527400	3D	605953283	605956699	6.06	103025.79
TraesCS5A02G444600	5A	624710650	624713923	6.36	102664.21
TraesCS5B02G526000	5B	685565831	685569284	8.25	103216.03
TraesCS7D02G053900	7D	28414579	28416907	8.31	78279.93
TraesCS5B02G454100	5B	626838041	626843887	5.98	78592.15
TraesCS1A02G016300	1A	8665006	8669951	5.39	75197.41
TraesCS2D02G070800	2D	29875384	29880239	7.94	78673.03
TraesCS7A02G062000	7A	30796784	30801041	5.74	80758.67
TraesCS6A02G377700	6A	598924318	598926783	5.95	81514.64
TraesCS6D02G394600	6D	467856066	467860898	5.95	105448.09
TraesCS6D02G210100	6D	296751801	296754290	5.76	84157.61
TraesCS6D02G056600	6D	27056856	27059250	6.92	81487.99
TraesCS2B02G580700	2B	768103607	768106123	6.68	74800.67
TraesCS5D02G374300	5D	447303949	447306105	6.32	65396.37
TraesCS2D02G070700	2D	29836691	29844564	8.06	79092.5
TraesCS1A02G340300	1A	530515730	530523983	6.72	83462.92
TraesCS5D02G256800	5D	362968139	362975213	8.04	81493.74
TraesCS6B02G100300	6B	78453112	78456063	5.74	81923.15
TraesCS7A02G563100	7A	733433253	733439394	5.82	108002.98
TraesCS4A02G448100	4A	714896540	714902419	8.4	81624.61
TraesCS1B02G021800	1B	9939530	9942269	6.16	72723.98
TraesCS3D02G527500	3D	605971468	605974837	5.6	98957.3
TraesCS6A02G377500	6A	598789628	598792184	5.84	82815.72
TraesCS4A02G026203	4A	18010331	18013889	7.75	76328.48

TraesCS3A02G034300	3A	19342872	19374762	6.26	73820.79
TraesCS6A02G377600	6A	598859239	598861843	5.65	83572.58
TraesCS2D02G378000	2D	482380071	482382529	5.97	79673.72
TraesCS5B02G273500	5B	458896741	458900165	6.48	73606.89
TraesCS3B02G295400	3B	474002444	474028365	6.08	72477.87
TraesCS7D02G241700	7D	205942378	205944687	5.98	79006.59
TraesCS2D02G509205	2D	601716189	601729664	8.07	82984.92
TraesCS6A02G225400	6A	423533078	423535555	5.71	82189.33
TraesCS3B02G595500	3B	818441522	818444036	6.31	80048.12
TraesCS1B02G050100	1B	29923154	29931444	7.87	75042.61
TraesCSU02G092400	Un	81993945	81996277	8.31	78638.45
TraesCS6B02G459300	6B	713822697	713827558	6.35	104857.87
TraesCSU02G035250	Un	30238391	30245379	5.86	93798.53
TraesCS5A02G261200	5A	475265303	475269422	6.02	82326.29
TraesCS1A02G011900	1A	6883652	6887141	6.51	83297.53
TraesCS4A02G482400	4A	737707108	737710711	8.93	83461
TraesCS6D02G010800	6D	4149643	4152417	6.35	80740.44
TraesCS5D02G452300	5D	500136721	500140169	5.87	101847.67
TraesCS3D02G494200	3D	587046159	587050702	6.35	119207.55
TraesCS5D02G374700	5D	447501303	447510051	5.82	117060.55
TraesCS1A02G058200	1A	38711994	38715061	6.15	73819.61
TraesCS2A02G071800	2A	31952804	31957974	6.65	77887.7
TraesCS6A02G376005	6A	598056417	598059222	5.79	83828.6
TraesCS5A02G084200	5A	109957043	109967255	5.56	78399.16
TraesCS5D02G043400	5D	42925913	42928461	6.61	83689.94
TraesCS1B02G075700	1B	58552822	58558289	6.19	74803.7
TraesCS6D02G028700	6D	11069121	11074665	6.12	103282.19
TraesCS6A02G000200	6A	103455	110107	5.32	100468.16
TraesCS2D02G046500	2D	17011036	17013413	6.48	81221.05
TraesCS3D02G046000	3D	17513600	17538118	6.26	71904.91
TraesCS2B02G059400	2B	28883113	28885490	8.01	81036.02
TraesCS3A02G006900	3A	7384784	7389062	5.09	82493.49
TraesCS5A02G249600	5A	464206324	464210946	7.61	81616.52
TraesCS6B02G056400	6B	36764447	36773780	5.71	75461.32
TraesCS6D02G395400	6D	468056247	468060799	5.8	82633.94
TraesCS3A02G522100	3A	738304030	738307517	6.81	106923.37
TraesCS6A02G008400	6A	3351576	3359760	6.27	102602.01
TraesCS2A02G047800	2A	18544624	18546971	8.42	81231.56
TraesCS2D02G002600	2D	2045570	2048979	5.98	79689.29
TraesCS5B02G449200	5B	621467718	621471226	6.25	102330.69
TraesCS5D02G520600	5D	541943276	541946160	5.92	83841.41
TraesCS6D02G069500	6D	34947606	34951802	7.85	83098.87
TraesCS6D02G361400	6D	452483344	452485884	5.81	82200.37
TraesCS5B02G452300	5B	625114904	625118686	5.59	77883.32
TraesCS6B02G073000	6B	49435434	49437836	6.68	81437.12
TraesCS6A02G361700	6A	591983346	591986913	5.11	78904.75
TraesCS5B02G089800	5B	115412946	115419618	5.6	78466.29
TraesCS4A02G391000	4A	667858921	667861337	5.61	79872.36
TraesCSU02G060500	Un	47477823	47480382	6.63	83685.13
TraesCS1B02G020200	1B	9489888	9494840	5.69	75163.47
TraesCS3A02G033400	3A	19275574	19307908	6.88	73831.42

TraesCS3B02G098900	3B	66416023	66420917	6.43	83972.72
TraesCS2B02G536500	2B	732038730	732042160	7.72	82905.58
TraesCS6D02G210200	6D	296824053	296826571	5.9	82304.68
TraesCS3D02G433100	3D	546385369	546389635	6.26	100565.67
TraesCS2A02G381400	2A	624941477	624944012	5.89	82217.34
TraesCS1B02G031700	1B	15517132	15519874	5.78	84868.76
TraesCS5B02G454700	5B	628694453	628698250	5.66	77956.42
TraesCS3A02G527700	3A	742223286	742228725	6.45	83282.23
TraesCS3D02G124200	3D	81811610	81816263	6.67	70255.17
TraesCS5D02G373400	5D	446875637	446878019	5.47	75565.27
TraesCS5D02G374600	5D	447424033	447432881	7.26	115931.87
TraesCS5D02G373700	5D	447140899	447143332	6.88	82028.03
TraesCS5A02G052900	5A	47956085	47965326	8.15	76698.8
TraesCS1D02G342300	1D	431959024	431969140	6.68	83643.14
TraesCS6B02G459600	6B	713906121	713910713	6.01	105332.88
TraesCS5B02G324400	5B	508675400	508678513	6.21	78686.13
TraesCS7A02G425900	7A	619031130	619036498	6.22	102968.21
TraesCS2D02G070600	2D	29740198	29745369	7.63	77599.45
TraesCS6B02G458200	6B	713458733	713462914	5.48	104971.19
TraesCS5A02G323800	5A	535519780	535522946	6.47	78865.55
TraesCS4A02G391600	4A	668160224	668162842	6.17	82887.58
TraesCS6A02G376200	6A	598154841	598157729	5.81	82211.42
TraesCS5B02G363100	5B	542191917	542201967	5.57	81635.19
TraesCS5A02G035300	5A	32710473	32712965	5.45	83016.85
TraesCS1A02G035700	1A	19891661	19894572	5.82	82144.66
TraesCS7D02G087200	7D	53496070	53498725	6.85	83300.33
TraesCS7B02G074949	7B	84440355	84454127	5.58	80754.22
TraesCS2D02G550900	2D	626592699	626595268	6.42	82268.01
TraesCS5B02G366900	5B	545575101	545577514	6.36	82172.15
TraesCS7A02G242800	7A	218236980	218240288	5.81	80853.02
TraesCS7D02G480100	7D	591007952	591011507	6.32	99260.68
TraesCS1D02G014900	1D	7073574	7077996	5.69	75059.33
TraesCS6D02G362200	6D	452906242	452908749	6.11	83607.97
TraesCS3A02G521900	3A	738236580	738240286	5.95	101595.47
TraesCS2B02G088100	2B	49840858	49846533	7.95	77386.43
TraesCS2D02G509200	2D	601708288	601711721	6.13	80734.5
TraesCS1B02G043400	1B	22822038	22825042	5.49	80363.31
TraesCS6B02G101400	6B	79716116	79720002	5.63	81561.48
TraesCS5D02G477400	5D	516637297	516640503	6.39	78261.61
TraesCS7D02G087000	7D	53431568	53434270	5.65	79570.19
TraesCS7A02G091500	7A	55880890	55883552	6.87	83354.29
TraesCS3D02G046900	3D	17642214	17670884	7.48	73507.9
TraesCS5B02G247400	5B	429093234	429097373	5.5	81881.82
TraesCS2B02G464100	2B	657850507	657854763	5.97	90787.04
TraesCS7A02G425700	7A	618681007	618684442	6.31	103769.92
TraesCS6A02G376500	6A	598189143	598191599	6.95	85001.12
TraesCS6B02G001500	6B	1002899	1011655	5.73	102401.37
TraesCS5D02G043500	5D	42930408	42932902	5.53	82882.47
TraesCS6B02G059800	6B	39367149	39369522	5.87	80924.51
TraesCS7A02G013700	7A	6297519	6300866	8.91	83819.35
TraesCS2D02G377900	2D	482348502	482351038	6.02	82356.25

TraesCS2B02G580400	2B	768057737	768060299	6.71	82464.27
TraesCS6B02G256300	6B	460274455	460276943	5.62	84150.45
TraesCS2A02G047600	2A	18422624	18424973	6.96	81084.96
TraesCS4A02G310100	4A	603034418	603038171	7.86	86144.57
TraesCS5B02G259500	5B	441664962	441671873	6.2	81828.03
TraesCS6D02G070300	6D	35491069	35494006	5.59	73255.7
TraesCS5B02G368200	5B	546564774	546569512	5.81	116895.93
TraesCS6B02G095800	6B	71866745	71870919	6.31	83126.33
TraesCS3A02G262100	3A	484990152	485011560	5.97	72078.34
TraesCS7A02G086200	7A	50233883	50243641	8.5	84504.31
TraesCS6D02G395700	6D	468146021	468153256	8.13	82835.53
TraesCS5A02G444700	5A	624722259	624725853	5.49	102278.66
TraesCS2D02G509500	2D	601963045	601971739	6.91	82702.99
TraesCS6B02G414000	6B	687177971	687180528	5.85	82670.9
TraesCS7D02G457100	7D	575593547	575597343	6.3	94774.73
TraesCS6B02G415100	6B	687954847	687957416	5.64	83867.03
TraesCS3B02G596500	3B	819363575	819368342	6.61	114453.35
TraesCS3B02G596900	3B	819629205	819634842	6.34	111076.19
TraesCS6B02G374000	6B	646741271	646745692	6.25	82956.51
TraesCS6B02G002495	6B	1934367	1938637	6.25	99144.68
TraesCS6B02G040700	6B	25254295	25257306	5.39	81614.58
TraesCS5A02G464700	5A	644097724	644105497	6.79	78486.11
TraesCS6B02G035500	6B	20960021	20963527	6.28	101877.01
TraesCS5D02G452200	5D	500131532	500135172	6.26	102075.99
TraesCS2D02G442000	2D	552192520	552195005	7.16	80929.48
TraesCS1A02G017700	1A	9232851	9236545	6.53	75430.92
TraesCS5A02G365300	5A	565488362	565490747	6.14	82012.98
TraesCS3B02G474400	3B	723456694	723460964	6.38	97809.69
TraesCS5D02G374500	5D	447392270	447396923	5.9	83373.12
TraesCS7D02G086900	7D	53424657	53427269	5.59	85060
TraesCS6D02G001400	6D	602989	606229	6.19	77743.12
TraesCS6B02G415000	6B	687873635	687877196	5.72	69576.51
TraesCS4A02G391100	4A	668083351	668085963	5.68	85047.02
TraesCS5B02G521800	5B	683992331	683995223	5.67	83939.3
TraesCS5D02G330600	5D	421986024	421989160	6.53	79278.98
TraesCS3B02G596300	3B	819279232	819285034	5.79	114595.45
TraesCS6D02G361000	6D	452468325	452470786	6.46	84173.05
TraesCS3A02G522900	3A	738690614	738694017	5.7	101836
TraesCS7A02G091400	7A	55425870	55428494	5.83	84949.75
TraesCS3A02G533100	3A	745897952	745900476	6.68	79833.05
TraesCS6B02G414900	6B	687731774	687734202	5.68	79418.61
TraesCS6B02G010600	6B	6337591	6344270	7.41	74853.28
TraesCS2A02G093100	2A	47308990	47311758	5.96	74220.31
TraesCS1D02G016200	1D	7429822	7445013	5.49	74918.99
TraesCS1D02G005000	1D	2436388	2448831	5.78	103548.45
TraesCS2A02G564700	2A	764770326	764777742	6.53	78626.5
TraesCS6A02G043900	6A	22953051	22955436	6.04	81734.44
TraesCS3B02G049100	3B	24911564	24944677	7.61	76449.23
TraesCS5A02G249400	5A	464176028	464189376	5.35	80112.43
TraesCS6B02G055400	6B	35720447	35723485	6.42	77077.06
TraesCS7B02G371500	7B	637568353	637590300	6.19	77624.61

TraesCS7B02G418300	7B	686647785	686651155	6.19	79491.4
TraesCS4A02G347600	4A	625485636	625488601	7.63	86850.97
TraesCS5D02G520800	5D	541952326	541955371	5.84	82857.47
TraesCS6B02G256200	6B	460206606	460209974	8.98	71179.81
TraesCS3D02G083900	3D	42631309	42636418	6.63	83649.46
TraesCS1B02G004100	1B	2354630	2357674	8.5	78674.56
TraesCS6B02G413900	6B	687132887	687135346	6.06	85210.09
TraesCS7D02G085600	7D	52695202	52697718	5.87	85018.82
TraesCS6D02G002500	6D	1508029	1517724	5.81	102903.78
TraesCS6D02G032300	6D	13598846	13601831	5.34	81410.44
TraesCS5D02G365500	5D	442694824	442697321	6.51	83023.1
TraesCS4D02G042300	4D	19752190	19755893	6.45	78086.47
TraesCS6B02G034300	6B	20698716	20706056	5.81	101697.31
TraesCS3B02G474500	3B	723463620	723467882	6.1	100324.39
TraesCS7B02G378200	7B	642562766	642568465	5.37	81324.05
TraesCS3A02G522300	3A	738431512	738435697	5.38	100361.24
