# Supplementary material for: Characterization of the WAK Gene Family Reveals Genes for FHB Resistance in Bread Wheat (Triticum aestivum L.)
Source: Int J Mol Sci. 2022 Jun 28;23(13):7157. doi: 10.3390/ijms23137157 (PMC9266398; doi:10.3390/ijms23137157)
Supplement: Supplementary file 1 [file ijms-23-07157-s001.zip › Table S2.pdf]

Table S2. Syntenic relationships between TaWAKs with other WAK genes in *H. vulgare* , *O. sativa* and *G.max.*

Wheat	*H. vulgare*
TraesCS2D02G442000.1	HORVU.MOREX.r3.2HG0194210.1
TraesCS2B02G464000.1	HORVU.MOREX.r3.2HG0194210.1
TraesCS5B02G324400.1	HORVU.MOREX.r3.5HG0499530.1
TraesCS2D02G508900.1	HORVU.MOREX.r3.2HG0204030.1
TraesCS7D02G053900.1	HORVU.MOREX.r3.7HG0642910.1
TraesCS5D02G268600.1	HORVU.MOREX.r3.5HG0490230.1
TraesCS5B02G247400.1	HORVU.MOREX.r3.5HG0488230.1
TraesCS5A02G084200.1	HORVU.MOREX.r3.5HG0442460.1
TraesCS5B02G089800.1	HORVU.MOREX.r3.5HG0442460.1
TraesCS5D02G096200.1	HORVU.MOREX.r3.5HG0442460.1
TraesCS5A02G261200.1	HORVU.MOREX.r3.5HG0490230.1
TraesCS5B02G259500.1	HORVU.MOREX.r3.5HG0490230.1
TraesCS6D02G032300.1	HORVU.MOREX.r3.6HG0543140.1
TraesCS5A02G249300.1	HORVU.MOREX.r3.5HG0488230.1
TraesCS7A02G242800.1	HORVU.MOREX.r3.7HG0678660.1
TraesCS5A02G323800.1	HORVU.MOREX.r3.5HG0499530.1
TraesCS7A02G013700.1	HORVU.MOREX.r3.7HG0635610.1
TraesCSU02G079300.1	HORVU.MOREX.r3.6HG0543140.1
TraesCS5D02G330600.1	HORVU.MOREX.r3.5HG0499530.1
TraesCS6B02G040700.1	HORVU.MOREX.r3.6HG0543140.1
TraesCS6A02G377500.1	HORVU.MOREX.r3.6HG0627720.1
TraesCS5D02G256700.1	HORVU.MOREX.r3.5HG0488230.1
TraesCS7A02G091100.1	HORVU.MOREX.r3.7HG0649350.1
TraesCS4A02G482400.1	HORVU.MOREX.r3.7HG0635610.1
TraesCS7D02G241700.1	HORVU.MOREX.r3.7HG0678890.1
TraesCS6B02G002495.1	HORVU.MOREX.r3.6HG0538930.1
TraesCS4A02G347600.1	HORVU.MOREX.r3.5HG0531690.1
TraesCS7D02G086900.1	HORVU.MOREX.r3.7HG0649350.1
TraesCS7A02G425700.1	HORVU.MOREX.r3.7HG0728630.1
TraesCS5B02G526000.1	HORVU.MOREX.r3.5HG0531690.1
TraesCS6A02G075500.1	HORVU.MOREX.r3.6HG0550560.1
TraesCS7D02G241500.1	HORVU.MOREX.r3.7HG0678660.1
TraesCS5D02G524800.1	HORVU.MOREX.r3.5HG0531690.1
TraesCS4A02G391000.1	HORVU.MOREX.r3.7HG0649350.1
TraesCS1D02G342300.1	HORVU.MOREX.r3.1HG0077000.1
TraesCS1B02G352600.1	HORVU.MOREX.r3.1HG0077000.1
TraesCS5A02G365300.1	HORVU.MOREX.r3.5HG0506000.1
TraesCS6B02G100300.1	HORVU.MOREX.r3.6HG0550560.1
TraesCS5B02G366900.1	HORVU.MOREX.r3.5HG0506000.1
TraesCS6D02G002500.1	HORVU.MOREX.r3.6HG0539050.1
TraesCS7D02G457100.1	HORVU.MOREX.r3.7HG0736720.1
TraesCS6D02G210200.1	HORVU.MOREX.r3.6HG0596130.1
TraesCS1A02G340300.1	HORVU.MOREX.r3.1HG0077000.1
TraesCS6B02G004000.1	HORVU.MOREX.r3.6HG0539050.1
TraesCS6A02G225300.1	HORVU.MOREX.r3.6HG0596150.1
TraesCS5D02G452200.1	HORVU.MOREX.r3.5HG0520120.1
TraesCS6B02G459100.1	HORVU.MOREX.r3.6HG0632470.1

TraesCS6A02G225400.1	HORVU.MOREX.r3.6HG0596130.1
TraesCS6B02G414900.1	HORVU.MOREX.r3.6HG0627720.1
TraesCS2A02G047600.1	HORVU.MOREX.r3.2HG0101670.1
TraesCS6B02G101400.1	HORVU.MOREX.r3.6HG0550560.1
TraesCS1D02G037200.1	HORVU.MOREX.r3.1HG0006210.1
TraesCS5D02G374500.1	HORVU.MOREX.r3.5HG0506000.1
TraesCS6D02G210100.1	HORVU.MOREX.r3.6HG0596150.1
TraesCS6D02G394600.1	HORVU.MOREX.r3.6HG0632470.1
TraesCS5D02G373700.1	HORVU.MOREX.r3.5HG0506000.1
TraesCS1B02G009600.1	HORVU.MOREX.r3.1HG0000540.1
TraesCS5D02G052800.1	HORVU.MOREX.r3.5HG0429150.1
TraesCS6A02G000200.1	HORVU.MOREX.r3.6HG0539050.1
TraesCS5B02G366400.1	HORVU.MOREX.r3.5HG0505950.1
TraesCS7B02G371300.1	HORVU.MOREX.r3.7HG0736720.1
TraesCS2D02G045900.1	HORVU.MOREX.r3.2HG0101670.1
TraesCS3B02G474400.1	HORVU.MOREX.r3.3HG0312090.1
TraesCS6D02G028700.1	HORVU.MOREX.r3.6HG0542390.1
TraesCS4A02G351600.1	HORVU.MOREX.r3.5HG0531280.1
TraesCS2D02G597700.1	HORVU.MOREX.r3.2HG0214050.1
TraesCS3D02G433100.1	HORVU.MOREX.r3.3HG0312090.1
TraesCS6B02G034300.1	HORVU.MOREX.r3.6HG0542390.1
TraesCS7A02G243000.1	HORVU.MOREX.r3.7HG0678890.1
TraesCS5B02G449200.1	HORVU.MOREX.r3.5HG0520120.1
TraesCS5A02G035200.1	HORVU.MOREX.r3.5HG0427440.1
TraesCS5D02G043400.1	HORVU.MOREX.r3.5HG0427440.1
TraesCS3D02G533100.1	HORVU.MOREX.r3.3HG0328800.1
TraesCS5A02G444600.1	HORVU.MOREX.r3.5HG0520120.1
TraesCS6D02G070300.1	HORVU.MOREX.r3.6HG0550050.1
TraesCS5D02G520500.1	HORVU.MOREX.r3.5HG0531280.1
TraesCS5B02G368200.1	HORVU.MOREX.r3.5HG0506170.1
TraesCS5A02G043600.1	HORVU.MOREX.r3.5HG0429140.1
TraesCS3D02G003900.1	HORVU.MOREX.r3.3HG0219240.1
TraesCS5D02G374600.1	HORVU.MOREX.r3.5HG0506170.1
TraesCS5D02G374300.1	HORVU.MOREX.r3.5HG0505950.1
TraesCS5D02G373400.1	HORVU.MOREX.r3.5HG0505950.1
TraesCS3A02G006900.1	HORVU.MOREX.r3.3HG0219240.1
TraesCS2A02G071800.1	HORVU.MOREX.r3.2HG0103900.1
TraesCS1D02G058800.1	HORVU.MOREX.r3.1HG0012290.1
TraesCS3B02G141500.1	HORVU.MOREX.r3.3HG0240070.1
TraesCS3D02G124200.1	HORVU.MOREX.r3.3HG0240070.1
TraesCS2D02G070600.1	HORVU.MOREX.r3.2HG0103900.1
TraesCS5A02G464700.1	HORVU.MOREX.r3.5HG0524050.1
TraesCS1A02G058200.1	HORVU.MOREX.r3.1HG0012290.1
TraesCS1B02G075700.1	HORVU.MOREX.r3.1HG0012290.1
TraesCS5D02G477400.1	HORVU.MOREX.r3.5HG0524050.1
TraesCS6D02G395400.1	HORVU.MOREX.r3.6HG0632750.1
TraesCS6B02G460900.1	HORVU.MOREX.r3.6HG0632750.1
TraesCS6A02G061200.1	HORVU.MOREX.r3.6HG0548400.1
TraesCS3D02G046000.1	HORVU.MOREX.r3.3HG0226150.1
TraesCS3D02G046900.1	HORVU.MOREX.r3.3HG0226150.1
TraesCS3A02G034600.1	HORVU.MOREX.r3.3HG0225430.1

TraesCS6D02G063400.1	HORVU.MOREX.r3.6HG0548400.1
TraesCS6B02G256200.1	HORVU.MOREX.r3.6HG0596150.1
TraesCS3A02G527700.1	HORVU.MOREX.r3.3HG0328800.1

**Wheat**	***O. sativa***
TraesCS2D02G442000.1	Os04g0598900
TraesCS2B02G464000.1	Os04g0598900
TraesCS5B02G247400.1	Os09g0471200
TraesCS6A02G376005.1	Os02g0807200
TraesCS6D02G032300.1	Os02g0111600
TraesCS6B02G413700.1	Os02g0807200
TraesCS5A02G249300.1	Os09g0471200
TraesCS7A02G242800.1	Os08g0501200
TraesCS6D02G361000.1	Os02g0807200
TraesCS6B02G040700.1	Os02g0111600
TraesCS5D02G256700.1	Os09g0471200
TraesCS4A02G347600.1	Os03g0841100
TraesCS7D02G086900.1	Os06g0142500
TraesCS5B02G526000.1	Os03g0841100
TraesCS7D02G241500.1	Os08g0501200
TraesCS5D02G524800.1	Os03g0841100
TraesCS4A02G391000.1	Os06g0142500
TraesCS7A02G091100.1	Os06g0142500
TraesCS5A02G365300.1	Os09g0561450
TraesCS7D02G136700.1	Os06g0170100
TraesCS6A02G225300.1	Os02g0623600
TraesCS6B02G256300.1	Os02g0811200
TraesCS6B02G414900.1	Os02g0807200
TraesCS6D02G210100.1	Os02g0811200
TraesCS2B02G464100.1	Os04g0598600
TraesCS5A02G035200.1	Os12g0614851
TraesCS5D02G043400.1	Os12g0615000
TraesCS5D02G043500.1	Os12g0614851
TraesCS3D02G046000.1	Os01g0136400
TraesCS3D02G046900.1	Os01g0136800
TraesCS3D02G046000.1	Os01g0136800
TraesCS6A02G376005.1	Os02g0811200
TraesCS6B02G414900.1	Os02g0811200
TraesCS6D02G361000.1	Os02g0811200

**Wheat**	***G.max***
TraesCS3D02G046900.1	KRH01725
