# Supplementary material for: Characterization of the WAK Gene Family Reveals Genes for FHB Resistance in Bread Wheat (Triticum aestivum L.)
Source: Int J Mol Sci. 2022 Jun 28;23(13):7157. doi: 10.3390/ijms23137157 (PMC9266398; doi:10.3390/ijms23137157)
Supplement: Supplementary file 1 [file ijms-23-07157-s001.zip › Table S3.pdf]

Table S3. Primers sequences used in this study.

Name	Forward (5'-3')	Reverse (5'-3')	Purpose
TraesCS7D02G086900	CTCTGGGAGCATCAGCATG	CGAATCTCACCCACAGTAAG	qRT-PCR
TraesCS7A02G091200	CGTCTGCTGGGAGCATCAG	TGTCCAGTACCCTAACTTCGG	
TraesCS7B02G148800	AGCAGGCGAGCTGTGCTCA	GCACGCCCCTGGGAATGGA	
TraesCS7A02G242800	GTTAGTGCACACCGTGGCTG	CACCGTGCCGCCAGGAATG	
TraesCS6B02G374000	ATGGCGTCCCACAACCACAA	ACGTTGCCATGGAATAGCGC	
TraesCS6A02G342000	CGTCCCACAAACCAGAAGCC	ACGTTGGAAAAGAACGGCGC	
TraesCS3B02G141500	GCCAGCAGATTCCTTCTGAT	GAGTTGTTCGCGTTCAGGAT	
TraesCS3D02G124200	AGCAGATTCCTTCGGATTG	CTAGAGTTGTTGGCGTTCAG	
TraesCS5A02G445700	AAGTCATCGGTGACTTGAGC	GACTGCCATCCCTGTCAGAT	
TraesCS5B02G458300	ATCCTACACGGTGATGTTAAAC	CAACTCGGTTGCTTTGTTCCA	
TraesCS5B02G454700	CGTACATCGCCGATGGATGC	GGTCTTCCTTCTCTCTTTGCG	
TraesCS3D02G046900	ATGGGTTTAGTGCTTCAAGG	CGAGACGTGCAACCATATAA	
TraesCS5A02G365300	GATCAGAGATGGCTGTGTCA	GTAGATGTCCCCTGTTTTGC	
TraesCS5B02G455500	ACGCCACAGTTGGCCTCTCG	TCATAGAACTCCCTGGTCTTC	
TraesCS5B02G454100	CAGCAACAGCTACTGTGTCA	CATATCCTCCCAACCTGTTC	
TraesCS3A02G034600	ATGTCTAGCATCGTCGTCGC	TGTGCGTCACCGCGTAGTCG	
TraesCS4A02G391100	CTGAAGGTTATGAGGGCAATC	GTCCTTCTCCTTCGCAAGCT	
TraesCS7D02G085800	ATGTGTCCAATGGCCCTGGA	CAGCCCGTAGAGCAAAGATAC	
TraesCS3D02G046000	GCCTGCCGTTCTTGGCCTCG	TCGGCGCGGCACACGCCGT	
TraesCS2B02G536500	CATCCTCGCAACGGTGACGG	GAGCAGCTTTGGTGGATGGC	
Tatubulin	GTGGAACTGGCTCTGGC	CGCTCAATGTCAAGGGA	
Ubi1300-5B02G454700-eGFP	AACGATAGCCGGTACCATGCCCACGCCCTCGCAATC	TGCTCACCATGGTACCGAGTCCAAGAACTTACGGTC	Subcellular localization
Ubi1300-3B02G141500-eGFP	AACGATAGCCGGTACCATGGCGAGACCGTTTTGCGG	TGCTCACCATGGTACCTACGTTTTGCTGCGGCTCCA	
Ubi1300-5A02G445700-eGFP	AACGATAGCCGGTACCATGCCCACGCCCTCCAAATC	TGCTCACCATGGTACCCCATCTACGAGACTTGTTCA	
Ubi1300-3D02G046900-eGFP	AACGATAGCCGGTACCATGGCTGCGCGTCTCATGCC	TGCTCACCATGGTACCGCATTTGTTGTTAGATAATC	

Ubi1300-2B02G536500-eGFP	AACGATAGCCGGTACCATGTCACAAGCAAAGCTCAT	TGCTCACCATGGTACCTCTTGGAATTTCAGATGATT
